# J. Payne, D.D.S., on Amalgam Plugs

**Published:** 1873-10

**Authors:** Burke Pillsbury

**Affiliations:** Duluth, Minn.


ARTICLE VII.—*J. Payne, D.D.S., on Amalgam Plugs.* By BURKE PILLSBURY, M.D., Duluth, Minn.

In the July number I find an article on "Poisoning from corrosive sublimate generated in the mouth from amalgam plugs in the teeth. By J. Payne, D.D.S." Your correspondent, it seems to me, takes a good deal of credit to himself for knowledge, which it is hardly to be expected that he, not being a medical man, should possess, and which in fact he does not seem to possess. When he makes such broad assertions he should offer some evidence of his acquaintance with the subject of which he treats, and while condemning an artificial filling in an old tooth as the cause of all earthly diseases, give us some satisfactory starting point. He mentions "twenty cases" he has had, "nearly all pronounced by physicians as consumption," leaving us to infer that they were otherwise, and so discovered by his superior skill and understanding. It would be interesting to his readers to know what means he and his rivals used in making their diagnoses. His symptoms are, "at times a very bad cough, eyes sunken, and haggard expression and deep blue or dark color under the eyes, invariably a metallic taste in the mouth, water flowing from the mouth in the night while asleep so as to wet the pillow, and in most cases extreme prostration"—any of which symptoms are common attendants of disease, and mark nothing in particular. What person has not, in perfect health, while dreaming of food, had saliva flow from the mouth more or less copiously, and not possessing a single amalgam filling?

Then his theory of bichloride of mercury being formed from the decomposition of the salts of the saliva, is pretty far-fetched, and would require all Liebig's skill in the demonstration. Chloride of sodium and potassium exist in the saliva in the proportion of *eighty-seven one-hundredths of one part in a thousand*, and the proportion of mercury that would escape, if at all, would be so infinitely minute that a microscope aided by the faith of a homœopath would not discover it. In addition to this, any one acquainted with chemistry knows that a chloride and mercury will not combine by any such simple process as contact and a little warmth.

